# Vectored immunoprophylaxis protects humanized mice from mucosal HIV transmission

**DOI:** 10.1186/1742-4690-9-S2-P42

**Published:** 2012-09-13

**Authors:** AB Balazs, J Chen, C Hong, S Ouyang, D An, D Baltimore

**Affiliations:** 1 California Institute of Technology, Pasadena, CA, USA; 2 University of California at Los Angeles, Los Angeles, CA, USA

## Background

Recently, a number of antibodies capable of broadly neutralizing HIV have been isolated from HIV infected patients, stimulating efforts to develop vaccines capable of eliciting their production in naive individuals. As an alternative to vaccination, we recently described vectored immunoprophylaxis (VIP) as an approach capable of generating high serum concentrations of a desired monoclonal antibody in mice following a single intramuscular injection of a specialized adeno associated viral vector (AAV). Mice that received VIP encoding b12 and VRC01 antibodies demonstrated long-term circulating antibody expression in serum, and VIP-treated humanized mice exhibited remarkable protection against high dose, intravenous challenge with CXCR4-tropic HIV. However, most human infections are initiated by transmission of CCR5-tropic strains through mucosal tissues.

## Methods

To measure the efficacy of VIP against clinically relevant strains, we humanized VIP-treated mice by adoptive transfer of peripheral blood mononuclear cells (PBMC) and challenged these animals with CCR5-tropic HIV strains including JR-CSF, as well as REJO.c, a transmitted molecular founder. To determine the ability of VIP to prevent mucosal transmission of HIV, we developed a repetitive intravaginal challenge model in VIP-treated BLT humanized mice that were challenged weekly with JR-CSF and monitored for infection.

## Results

PBMC humanized mice expressing either b12 or VRC01 were protected from intravenous challenge with JR-CSF. In contrast, the b12-resistant REJO.c strain readily infected PBMC humanized mice expressing b12 antibody, while mice expressing VRC01 demonstrated nearly complete protection following challenge. Intravaginally challenged BLT animals expressing a luciferase negative control protein all became infected over the study period while a majority of animals expressing VRC01 had no detectable HIV infection despite fourteen intravaginal challenges with JR-CSF.

## Conclusion

VIP is capable of protecting humanized mice from challenge by diverse HIV strains and can substantially inhibit mucosal transmission. These findings warrant continued development of VIP as a novel approach for HIV prevention in humans.

